# Anomalous Coronary Artery Origin from Pulmonary Artery and Coronary Fistulas: A Review About Coronary Steal Phenomenon

**DOI:** 10.3390/children13030424

**Published:** 2026-03-19

**Authors:** Mario Giordano, Martina Evangelista, Enrico Piccinelli, Sara Moscatelli, Domenico Sirico, Giovanni Meliota, Maria Giovanna Russo, Gianfranco Butera, Biagio Castaldi, Massimo Chessa, Gabriele Rinelli, Silvia Favilli

**Affiliations:** 1 Pediatric Cardiology Unit, University of Campania “Luigi Vanvitelli”, “Ospedali dei Colli”, 80131 Naples, Italy; 2 Pediatric Cardiology and Grown-up Congenital Heart Disease Unit, “IRCCS Policlinico San Donato”, 20097 Milan, Italy; 3 Pediatric Cardiology Unit, Pediatric Department, Buzzi Children’s Hospital, 20154 Milan, Italy; 4 Cardiology, Cardiac Surgery and Heart Lung Transplantation, Ern Guard Heart, Bambino Gesù Hospital and Research Institute, IRCCS, 00165 Rome, Italygianfranco.butera@opbg.net (G.B.);; 5 Centre for Inherited Cardiovascular Diseases, Great Ormond Street Hospital, London WC1N 3JH, UK; 6 Institute of Cardiovascular Sciences, University College London, London WC1E 6BT, UK; 7 Pediatric Cardiology Unit, Royal Brompton Hospital, London SW3 6NP, UK; 8 Pediatric Cardiology and Congenital Heart Disease Unit, “ASST Papa Giovanni XXIII”, 24127 Bergamo, Italy; domenico.sirico@asst-pg23.it; 9 Pediatric Cardiology Unit, Giovanni XXIII Pediatric Hospital, 70126 Bari, Italy; giovanni.meliota@policlinico.ba.it; 10 Pediatric Cardiology Unit, Department of Women’s and Children’s Health, University Hospital of Padua, 35128 Padua, Italy; biagio.castaldi@unipd.it; 11 Department of Cardiovascular Diseases, “Vita Salute” San Raffaele University, 20132 Milan, Italy; 12 Cardiology Unit, Meyer Children’s Hospital IRCCS, 50139 Florence, Italy

**Keywords:** coronary artery, ALCAPA, ARCAPA, ATCAPA, coronary fistulas

## Abstract

Anomalous coronary artery origin from pulmonary artery and coronary–pulmonary fistulas are the major causes of ischemic cardiopathy in children. Both anomalies are characterized by a connection between a higher and a lower pressure chamber causing coronary steal. However, several mechanisms and associated lesions may be responsible of the different presentations of the “coronary steal phenomenon”. The aim of this review is to highlight the different embryology, anatomical features, clinical presentation, and the diagnostic and therapeutic strategy of these coronary anomalies, despite their similar pathophysiology.

## 1. Introduction

Coronary artery (CA) anomalies are classified in four different types: of origin and course, intrinsic CA anatomy, CA termination, or associated with anomalous anastomotic vessels [1,2,3]. Anomalous origin of CA from the pulmonary artery (PA) and coronary-artery fistulas are both characterized by the coronary steal phenomenon due to left-to-right shunt with consequent myocardial ischemia (MI).

The aim of this review is to highlight the features of these anomalies and their physio-pathological basis.

## 2. Anomalous Origin of Coronary Artery from Pulmonary Artery

### 2.1. Embryology, Anatomy, and Classification

The CA formation derives from the ingrowth of a capillary plexus into the aortic sinuses. The migration and differentiation of the primitive cells into endothelial cells, vascular smooth muscle cells, and fibroblasts are mediated by several transcription factors (HIF-1α, HIF-β, VEGF-A, VEGF-B). HIF-1 (α/β dimer) is a regulator of hypoxia adaptation. VEGF-A and VEGF-B promote angiogenesis, endothelial cell migration, and vessel maturation. A chronic anomalous expression of these factors may influence the development of the coronary arteries with a consequent anomalous origin [4]. During fetal life, left and right CA are connected to the anterior part of the left (1) and right (4) leaflet of the common arterial trunk (AT); therefore, both CAs will arise from the aorta after AT septation. Two main hypotheses might explain the anomalous origin of CA from the PA (ACAPA): both or one of the primitive CAs are connected more posteriorly to the AT and are consequently connected to the PA after the AT septation; otherwise, there is an anomalous AT septation [5].

ACAPAs may be categorized as anomalies of origin and course, along with anomalous aortic origin of CA from the opposite coronary sinus or from an improper location (e.g., high, low, or commissural takeoff of the CA) [1,2,3].

Multiple morphological variants of ACAPA exist. Based on the CA involved, they may be grouped as follows: (1) anomalous left coronary artery (LCA) from the PA (ALCAPA), (2) anomalous right coronary artery (RCA) from the PA (ARCAPA), (3) anomalous total origin of CAs from the PA (ATCAPA), (4) anomalous origin of circumflex artery from the PA (ACxAPA), and (5) anomalous origin of left anterior descending artery from the PA (ALADAPA).

ACAPA may be classified also based on the site of origin of the anomalous CA. Anomalous LCA originating from the right or the posterior sinus of valsalva of the PA is the most common type of ALCAPA. LCA originating from the left-sided wall of the PA is a further less frequent presentation. LCA arising from the right PA is often associated with an intramural course through the aortic wall and is the rarest form [6,7,8]. No clear data regarding the site of origin of RCA in ARCAPA are present in the literature.

ATCAPA is usually characterized by a single orifice from the PA; dual-orifice ATCAPA is a rare presentation. The origin from the right or left PA is the rarest [9].

### 2.2. Epidemiology

ACAPA is generally diagnosed by one year of age. As the main study regarding the incidence of CA anomalies is based on a cohort of patients undergoing CA angiography due to ischemic symptoms, the bias of selection is obvious. Accordingly, ARCAPA anatomy was found in 0.002% of patients. The incidence of ALCAPA is 1/300,000 live births, representing 0.24% to 0.46% of congenital heart disease [9,10,11]. Only a minority of cases of ATCAPA are present in the literature [12,13]. A noticeable predominance of ALCAPA in females (>2:1) has been reported, compared with a slight predominance in males for ARCAPA [12,14].

Sudden cardiac death (SCD) among untreated adults with ALCAPA, according to an early autoptic study, occurs at an average age of 35 years [15,16]. Nevertheless, with the increasing diagnostic rate of adult ALCAPA cases, the true association between ALCAPA and SCD may be lower than estimated, especially among older patients.

### 2.3. Pathophysiology and Clinical Manifestations

Different forms of ACAPA share the same pathophysiology of the coronary steal phenomenon but present various clinical onsets. An exception is made for ATCAPA, which has a different pathophysiology responsible for its clinical manifestation.

ALCAPA may be classified as “infantile presentation” or “adulthood presentation”. The infantile presentation is characterized by early onset of symptoms; ischemic cardiopathy usually develops within 3–4 months of life. A progressive drop in pulmonary vascular resistance (PVR) is responsible for the onset of the coronary steal phenomenon: inadequate collateralization leads to suboptimal supply of the LCA field and subsequently MI. MI causes direct left ventricle (LV) damage with dilatation and hypokinesia (i.e., “acute ischemic cardiopathy”). The overall consequence is low cardiac output with systemic hypoperfusion (up to cardiogenic shock) and increased LV and left atrium (LA) pressure provoking pulmonary congestion. Both problems are worsened by mitral regurgitation (MR) which is due to primary (papillary disfunction) and secondary (LV dilatation) mechanisms [17].

In the infantile presentation, a large persistent ductus arteriosus may hide the clinical manifestations of ALCAPA since it contributes to keeping high PVR (with a consequent lowering of the coronary steal). Other extra-cardiac causes of high PVR (e.g., bronchial dysplasia, pneumonia, persistent neonatal pulmonary hypertension, and Down syndrome) have the same mechanism. In these circumstances, an early diagnosis is essential to avoid therapeutic delays.

The adulthood presentation is characterized by significant collateralization between RCA and LCA. The large collaterals supply the LCA field with protection from coronary steal. However, not every segment has adequate collateralization; therefore, some areas receive insufficient perfusion, causing segmental wall motion anomalies (hypo-, a-, or dyskinesis area). The worst peak longitudinal strain (LS) is present in the basal lateral LV segment, but abnormal LS is found in the whole anterior and lateral wall, basal and middle segments of the posterior wall, and the apex of the LV [18]. Segmental ischemic damage contributes to progressive LV dilatation and insures “chronic ischemic cardiopathy”, with symptoms such as angina and/or dyspnea on effort. Chronic MI is the main cause of myocardial fibrosis, and larger myocardial fibrosis means higher risk of SCD, as confirmed by MRI [19,20,21,22,23].

MR is a characteristic of adult presentation and may be caused by scarring and calcification of papillary muscles, endocardial fibroelastosis, LV and mitral valve ring dilatation, and alteration of the papillary muscles’ geometry [24]. The coronary steal phenomenon is associated with left-to-right shunt, which contributes to left-sided chamber dilatation with worsening of MR.

**Figure 1:**
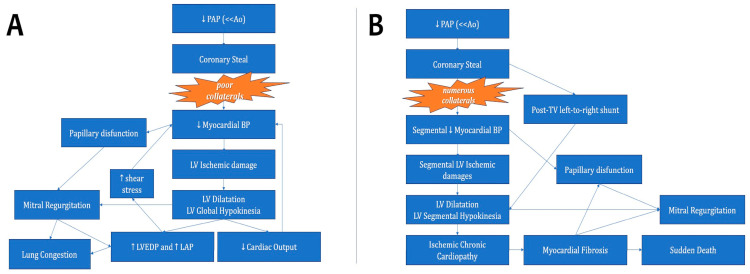
The algorithm summarizes the pathophysiologic cascades of both infantile (**A**) and adulthood (**B**) presentation of ALCAPA. Abbreviations: Ao: aorta; BP: blood perfusion; LAP: left atrial pressure; LV: left ventricle; LVEDP: left ventricle end-diastolic pressure; PAPm: mean pulmonary arterial pressure.

The pathophysiologic cascades of both infantile and adulthood presentation of ALCAPA are summarized in Figure 1.

The pathophysiology of ARCAPA, ACxAPA, and ALADAPA is like the “adulthood presentation” of the ALCAPA, since the less extensive myocardial field supplied by the “anomalous artery” allows the development of significant collateralization. Therefore, the infantile presentation of ARCAPA, ACxAPA, and ALADAPA is very rare [25].

In ATCAPA, there is no vascular connection between the aorta and the PA, no collateralization develops; thus, there is no “coronary steal phenomenon”. However, after the PVR fall, the mean pulmonary pressure is not able to support the coronary artery with consequent acute MI (as in ALCAPA’s infantile presentation). ATCAPA presents increased severity of clinical presentation and greater anticipated timing of surgery [9].

### 2.4. Diagnosis

The diagnosis of ACAPA is challenging. The electrocardiogram may present atrial enlargement (left atrium or biatrial), a typical ischemic anomaly of QRS or ST-T segment (abnormal deep and wide Q waves in leads I and aVL). However, in asymptomatic patients with ARCAPA, ALADAPA, or ACxAPA, these alterations may be trivial or absent.

The diagnosis is usually confirmed by trans-thoracic echocardiography (TTE). In ALCAPA, TTE may detect three different elements:

- direct signs;
- typical indirect signs;
- atypical indirect signs.

The direct signs are the detection of retrograde blood flow in the LCA with associated shunt into the PA. The typical indirect signs are large coronary collateralization and significant RCA dilatation (RCA/aorta ratio > 0.12) [26]. These signs are more evident in adulthood than infantile presentation [26,27]. The atypical indirect signs are LV dilatation, LA dilatation, segmental or global kinesis anomalies, MR, endocardial fibroelastosis, scarring, and calcification in the LV papillary muscles [24,26,27]. Usually, the infantile type presents global hypokinesis of the LV versus the segmental kinetic anomalies of the adulthood presentation [18,26]. TTE based on direct and indirect typical signs has a diagnostic accuracy of 90.9% [28]. The limitations of echocardiographic diagnosis are due to the challenges in differentiating true signals from blood flow artifacts when the ultrasounds analyze small size vessels such as the coronary arteries. For these reasons, echocardiography is very helpful when there is a diagnostic suspicion, but advanced imaging is often necessary to confirm diagnosis [28,29].

**Figure 2:**
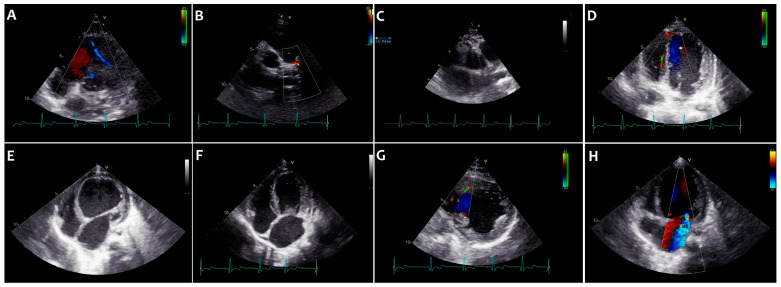
Echocardiographic direct signs: retrograde blood flow in the LCA (#) (**A**) with associated shunt into the PA (circle) (**B**). Echocardiographic typical indirect signs: significant RCA dilatation (**C**) and large coronary collateralization (**D**,**G**). Echocardiographic atypical indirect signs: LV dilatation (**E**), LA dilatation (**F**), and mitral regurgitation (MR) (**H**).

The main echocardiographic findings of ALCAPA are summarized in Figure 2.

The echocardiographic diagnosis of ARCAPA, ALADAPA, and ACxAPA is usually more complex. The dilatation of the original CAs and the identification of important collateralization allow us to suspect their presence. However, the diagnosis is confirmed by the detection of retrograde flow within the anomalous CA.

TTE diagnosis of ATCAPA is the most complex. Neither RCA/LCA dilatation nor obvious collateralization are present. The CA arising from the PA has an antegrade flow (no retrograde). However, patients present with acute ischemic cardiopathy with severe dilatation and global hypokinesis of LV. The detection of a neonatal/infantile dilatative hypokinetic cardiopathy with significant MR associated with a lack of view of the aortic coronary ostia should arouse the suspicious of ATCAPA, which will then be confirmed by another imaging technique (cardiac catheterization, angio-CT scan, or cardiac MRI) [29].

Angio-CT scan is the gold-standard technique used to diagnose ACAPA, since it has the best anatomic details [30]. Cardiac MRI provides information about myocardial fibrosis and may be useful in predicting a significant improvement in kinesis after surgical repair [19,31]. Brown et al. [31] demonstrated that sub-endocardial late hyper-enhancement is associated with extensive fibrosis and poor prognosis after surgical correction.

**Figure 3:**
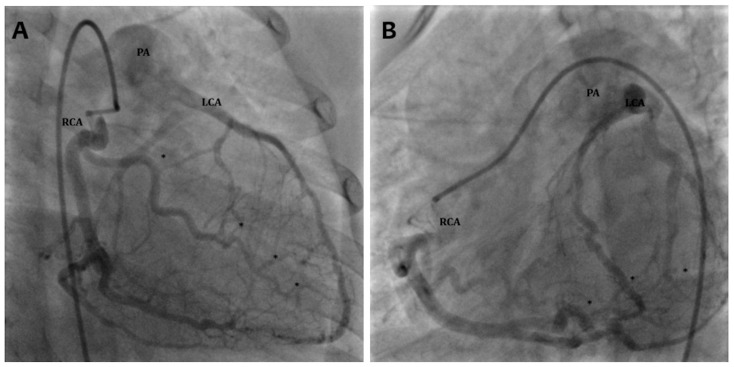
Selective right coronary artery angiography in adulthood presentation ALCAPA. Both the right (**A**) and left (**B**) anterior oblique view highlight a dilatated right coronary artery (RCA) with numerous coronary collaterals (*) responsible of a retrograde left coronary artery (LCA) opacification draining into the pulmonary artery (PA).

Cardiac catheterization allows an anatomic diagnosis of ACAPA; however, it is an invasive exam and should be adopted only when angio-CT scan is not available (Figure 3).

In asymptomatic patients with ARCAPA, ALADAPA, or ACxAPA, it is crucial to detect inducible MI for subsequent surgical repair. Scordino et al. [32] suggested adopting both an exercise stress and a dipyridamole stress test to explore inducible ischemia. The exercise stress test reproduces MI due to stress triggers, which are more common in the daily life, whereas dipyridamole mimics coronary steal due to vasovagal triggers (with a reduction in both systemic arterial pressure and vessel resistance in the collateral-dependent myocardium).

### 2.5. Treatment

The mortality rate for ALCAPA is up to 90% and may increase up to 100% for ATCAPA [33,34]. Thus, the indication for surgical correction is the diagnosis itself. The goals of surgical repair are to eliminate coronary steal and to establish a two-vessel aortic-derived CA system. Surgery has shown excellent short- and long-term results in restoring dual CA circulation [33,34,35,36]. Indeed, surgery is life-saving in neonates and in adults, hence the indication for urgent surgical repair remains regardless of age or degree of coronary collateralization. In truth, adults with well-represented collateral circulation continue to have significant left-to-right shunt and poor coronary reserve due to the coronary steal phenomenon, which predisposes them to MI, arrythmia, and SCD [14,37,38]. However, Lotman et al. [39] suggested a conservative approach to “late adult presentation” of ALCAPA. The label “late adult ALCAPA” is adopted with late diagnosis of ALCAPA (>60 years-old), mild or no symptoms, good collateralization development, and dilated and dominant RCA. In these circumstances, the risk of SCD appears to decline. In these patients, percutaneous closure of the ALCAPA ostium could be a valid “low-risk” approach to protecting from coronary steal [40].

ARCAPA, ACxAPA and ALADAPA have been recognized as less malignant because of less myocardial field interested by coronary steal. The ESC guidelines suggest surgical repair of ARCAPA in symptomatic patients (class I) and in asymptomatic patients with ventricular dysfunction and/or MI due to CA anomalies (class IIa) [41].

Different approaches have been proposed to restore dual coronary circulation, from the ligation of the anomalous CA to coronary artery bypass grafting (CABG) in different variations, but intrapulmonary baffle (Takeuchi-type repair) and direct CA reimplantation to the aorta remain the best options [35,42,43,44,45]. The crucial element guiding the decision between these two procedures is the position of the anomalous CA: direct anastomosis with mobilization of the CA ostium should be chosen when LCA originates from the right-sided PA wall or the posterior PA wall, and the Takeuchi-type repair should be preferred in case of origin from the left-sided wall of the PA [43,45].

In adult patients with established CA disease, CABG with ligation of the anomalous CA should be considered [44]. In infantile presentation, the small size of coronary artery does not allow this surgical technique.

The early mortality rate after corrective surgery of ACAPA ranges from 0% to 16% and is related to preoperative LV dysfunction. It may be reduced by careful use of postoperative cardiac support techniques, and its use ranges from 25% to 36% [46,47,48,49].

The management of MR is controversial. MR improves along with LV function, but recovery may be incomplete; therefore, mitral valve surgery is not strongly indicated at initial surgery, but it should be considered in specific cases with low potential for recovery. These latter cases may be grouped according to the presence of severe MR with relatively well-preserved LV function and fibrosis of the papillary muscle or mitral valve dysplasia [50]. Mitral valve surgery during ALCAPA correction prolongs the ischemic time and could be potentially harmful [47,50,51,52], even if this is not associated with higher mortality [53].

### 2.6. Complications and Follow-Up

Early diagnosis and prompt surgical intervention guarantee low mortality. Still, the risk of long-term complications and chronic sequelae due to primitive MI remains [13,45].

Postoperative complications are rare; the worst are MI due to stenotic anastomosis, aneurismatic dilatation of the CA anastomosis, iatrogenic aortic valve or pulmonary valve lesions, and RV outflow tract obstruction after Takeuchi repair [13,43,44,45,54].

During long-term follow-up, both CA ostium reimplantation and the Takeuchi procedure improve LV ejection fraction and LV volumes [55], but the diastolic and longitudinal systolic function of all chambers remain impaired, especially in the LCA region [18,56].

Takeuchi repair is associated with a higher risk of re-intervention, heart transplantation, or death [57]. Takeuchi’s baffle lesions (aneurysms, leakages, or stenosis) and pulmonary supra-valvular stenosis are the main complications related to Takeuchi repair [58].

After ACAPA repair, long-term follow-up should be focused on assessing the remaining risk of SCD [59]. Lifelong risk of SCD persists even after successful surgical correction and is directly related to the entity of myocardial fibrosis. The main cause of SCD is malignant ventricular arrythmias, which are a direct consequence of macro re-entrant circuits caused by myocardial fibrosis. In this setting, an electrophysiological study may be helpful in detecting a ventricular re-entrant and ablating this arrhythmogenic substrate [54,59,60].

Regular clinical follow-up with EKG and first-line imaging with TTE are of the utmost importance. Clinical evaluation should focus on new or progressive signs and symptoms of heart failure and MI. EKG should be assessed to highlight new ischemic modifications, and TTE should be focused on assessing post-operative complications, myocardial function, and modification of MR [54,61].

Cardiac CT scan and/or MRI is recommended as second-line imaging techniques [50,51,62]. Cardiac CT scans are helpful in evaluating CA anastomosis and, in Takeuchi-type repair cases, the baffle through its length. If cardiac CT scan shows any abnormalities or a doubtful result, a coronary angiogram should be performed. Coronary angiography is the gold-standard technique used to investigate patients with typical or atypical symptoms of MI, especially after CABG with ligation of the CA or after ligation of the anomalous CA [54]. Cardiac MRI may be helpful in risk stratification because it facilitates assessment of myocardial function and fibrosis; whenever there is suspicion of MI, it should be associated with stress MRI to test viability [19,31].

## 3. Coronary Artery Fistulas

### 3.1. Embryology, Anatomy, and Classification

Congenital CA fistulas (CAFs) originate from the persistence of embryonic intramyocardial trabecular sinusoids. Generally, these structures narrow in adulthood and persist as thebesian vessels. If their obliteration fails, an abnormal connection persists between the CA and a cardiac chamber [63,64].

Thus, CAFs are abnormal connections between one or more CAs and major thoracic vessels or cardiac chambers, which bypass the capillary bed. Fistulas that terminate into the heart are known as coronary–cameral fistulas, while those draining into a vein are known as coronary arterio-venous fistulas. Most commonly, CAFs drain to the right heart or great vessels such as the superior vena cava, coronary sinus, PA, and pulmonary veins [64,65], while fistulas draining to the left heart chambers are rare [66,67]. Most CAFs are congenital and may occur in isolation or in association with other congenital heart diseases. In rare cases, CAFs are acquired or iatrogenic, often related to cardiac surgery, interventional procedures, infection, or chest trauma [68,69,70,71].

Multiple classifications have been proposed. CAFs can be described as small, medium, or large according to their diameter (<1, 1–2, or >2 times the largest diameter of the CA not feeding the coronary fistula, respectively) [72]. CAFs can also be classified according to their origin. In proximal-type fistulas, the proximal coronary tract before the origin of the fistula is dilated, while the distal end is normal. In distal-type fistulas, the entire length of the CA is dilated with normal branching and continues as a fistula in the heart or a vessel.

### 3.2. Epidemiology

CAFs are rare in the general population, and the true incidence is unknown because approximately half of patients are asymptomatic. The prevalence roughly ranges from 0.05% to 0.9% of patients undergoing coronarography or CT scan [73,74,75,76].

Most CAFs are single (74–90%), while multiple fistulas range from 10% to 16%, with both CAs involved in 5% of the cases [77]. More than 90% of CAFs drain to the right chambers, originating from the RCA or LCA in equal number [78].

### 3.3. Pathophysiology and Clinical Manifestations

The pathophysiology of CAFs is determined by the size, length, tortuosity, exit point and connection of the fistula. In fistulas draining into the right heart, blood flows through the lower-resistance fistula pathway, avoiding the higher resistance of myocardial arterioles and capillaries. Thus, there is a potential “coronary steal phenomenon” due to this left-to-right shunt. In the less common cases of CAFs draining in the left heart chambers, there is a run-off similar to aortic valve regurgitation. Persistent high flow causes progressive dilatation and the formation of an aneurysm of the fistula and the native CA [79,80,81].

Patients with small CAFs are often asymptomatic, and fistulas may close spontaneously during follow-up. The diagnosis of CAFs is usually incidental during non-invasive cardiac imaging or coronary angiography. Usually, symptoms occur later in life with fatigue, exertional dyspnea, and heart failure. Larger and hemodynamically significant fistula can manifest with angina, MI, and myocardial infarction due to coronary steal. Aneurysm rupture is possible, resulting in hemopericardium. Also, arrhythmias such as atrial fibrillation and ventricular arrhythmias have been described in association with CAFs. In rare cases, endocarditis/endarteritis have been associated with fistulas, with an incidence of 1–12% in different reports [82,83,84].

### 3.4. Diagnosis

Clinical examination usually reveals a continuous murmur at the left lower sternal border. EKG is often normal but may reveal right or left ventricular volume overload in large CAFs. An ischemic pattern is possible if coronary steal is present.

Chest X-ray may confirm cardiomegaly and pulmonary plethora in cases of congestive heart failure. Sometimes, an enlarged cardiac silhouette may reveal aneurysmal dilation of the feeding coronary artery [85,86].

TTE generally shows CAFs as an incidental finding but is important in describing the size, number, pathway and drainage of the fistula. Moreover, it allows the detection of chamber dilatation, signs of MI, and associated anomalies.

**Figure 4:**
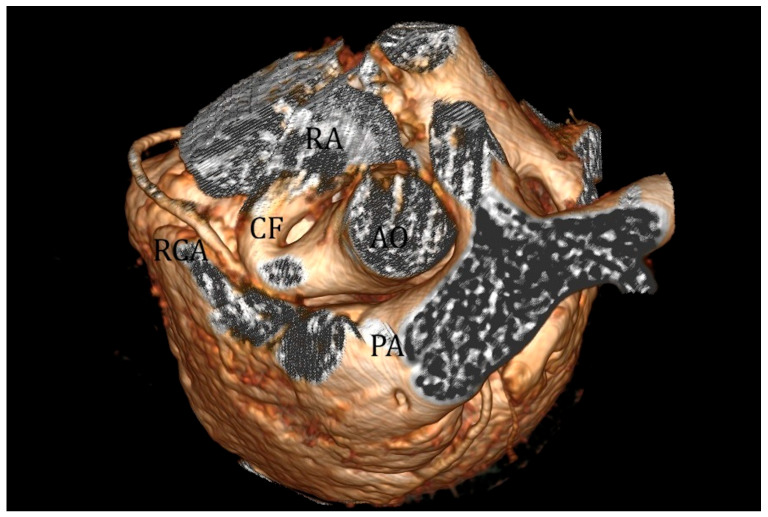
Angio-CT scan three-dimensional view of a large coronary fistula (CF) between the right coronary artery (RCA) and the right atrium (RA). Abbreviations: AO: aorta; PA: pulmonary artery.

Advanced imaging with cardiac CT scan and 3D reconstruction can accurately delineate the course of coronary fistulas, making it an essential tool in planning interventional or surgical procedures (Figure 4) [87]. Angiography remains the best imaging modality for CAF diagnosis and management.

### 3.5. Treatment

Small fistulas in asymptomatic patients are usually incidental findings and need conservative management with continued monitoring in children as they can grow during follow-up. The main indications for CAF closure are the presence of a significant left-to-right shunt that causes ventricular overload and/or signs of MI. Medium-to-large-size CAFs should be closed early in the course of the disease, since closing larger fistulas later in life exposes individuals to a higher risk of myocardial infarction, especially in cases of significant shunt [72,88,89,90].

**Figure 5:**
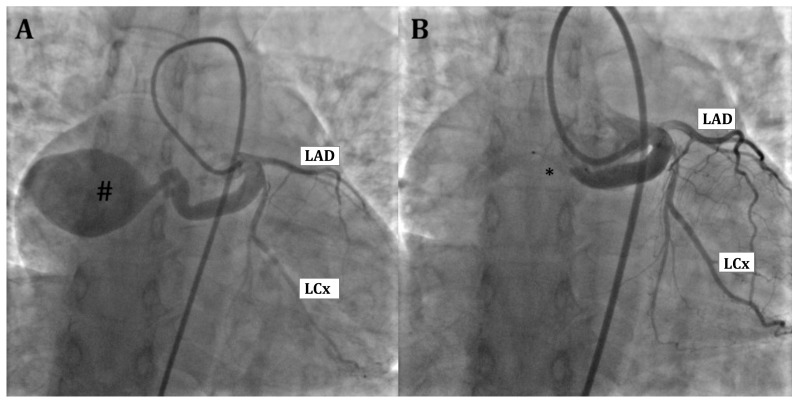
Selective left coronary artery angiography. A large coronary fistula (#) between the main stem and the right atrium (**A**) that has undergone percutaneous closure using an Amplatzer Vascular Plug IV 8 mm (*) (**B**). Abbreviations: LCx: left circumflex artery; LAD: left descending anterior coronary artery.

For percutaneous closure, several devices, coils, and vascular occluders have been used to close CAFs (Figure 5) [91]. Sometimes, multiple coils or devices are needed to abolish residual shunt, reducing the risk of fistula recanalization. In rare cases, a stent graft can be used to exclude web-like CAFs or fistulas with multiple origins. Transcatheter closure of acquired CAFs is also possible with high success rates [92]. Percutaneous closure of CAFs can be performed with a trans-arterial or a trans-venous approach. In the first case, the fistula is approached and closed, cannulating the native CA feeder, while the trans-venous approach often needs the creation of an arteriovenous loop to close the fistula at its exit site. The trans-arterial approach is generally preferred for proximal CAFs, due to the short navigation into the native CA. Another consideration, especially in small children, is the size of the introducer needed to deploy the appropriate device, which may influence the decision as to which vascular approach to use. A trans-venous approach may be preferred for distal CAFs, potentially reducing the risk of damaging the feeding vessel [72]. Creating an arteriovenous loop by snaring and exteriorizing a wire may help in terms of support and device delivery in cases of tortuous fistula and multiple small fistula exits. After fistula closure, different anti-platelet/anticoagulation regimens have been proposed; some prescribe anti-platelet therapy for six months or for life, and other centers suggest anticoagulation in aneurysmal and “low-flow” CAFs [93,94].

In proximal fistula closure, the procedure aims to abolish the flow of the fistula confluence or all its feeders. In some cases, this can be challenging, as there are several feeders and connections to the vascular structures. Sometimes, multiple plexiform fistulas are present, which makes their closure impossible. Different devices, coils, and micro-coils can be used. In rare cases, a covered stent can exclude the fistula.

Procedural planning is essential in distal CAF closure. It is fundamental to understand the size of the fistula and the aneurysmal native CA, the takeoff, the number and distribution of CA branches, and the outlet of the fistula. The potential thrombus in the aneurysmal native CA should be taken into account in decision making, and anticoagulation management should be accurately planned [80]. In cases of severely dilated CA, CAF surgical closure and CABG can be considered [72].

For surgical closure, from the first description of CAF’s successful surgical closure by Bjork and Crafoord in 1947 [95], different surgical techniques have been reported, with the aim of obliterating the fistula and preserving myocardial coronary flow.

The most common surgical option is epicardial ligation, especially in the case of distal CAFs without coronary branching distal to the fistula. An occlusion test with EKG monitoring is often required before definitive closure to exclude MI.

The trans-coronary approach is preferred when the native CA is aneurysmatic and it is necessary to cut the aneurysmal tract [96]. Various surgical techniques can be applied, such as isolating or resecting the aneurysm and reconstructing the coronary course (for instance, by using an interposition graft or by maintaining distal coronary flow by concomitant CABG).

Long-term follow-up is essential due to the possibility of postoperative recanalization, persistent dilatation of the CA and ostium, thrombus formation, calcification, arrhythmias, and myocardial infarction. Anti-platelet therapy with aspirin after trans-coronary CAF closure is suggested due to direct vascular trauma [97].

### 3.6. Complications and Follow-Up

CAF percutaneous closure procedures remain challenging even in specialized tertiary cardiac centers, with procedural success mainly depending on favorable anatomy and ranging from 80 to 90%. The potential risks range from iatrogenic damage to the CA (through rupture, dissection, thrombosis, or stenosis) to device embolization, especially in large fistulas [88,98].

Myocardial infarction can occur due to manipulation in the native CA or device thrombosis and may even occur after a successful procedure due to thrombus translocation from the proximal part of the occluded fistula. Surgical repair can also be complicated by myocardial infarction [84,99]. Finally, fistula recanalization is possible despite being rarely described and often not hemodynamically significant. El-Sabawi B et al. performed a follow-up angiography after a median time of 423 days from successful CAF closure in 22 patients, showing a large flow from recanalization in 4 patients [88].

## Data Availability

No new data were created or analyzed in this study.
